# Evaluating the feasibility of a tele-diagnostic auditory brainstem response service in a rural context

**DOI:** 10.4102/sajcd.v71i1.1020

**Published:** 2024-07-31

**Authors:** Divhanani Sithi, Samantha M. Govender, Thembelihle S. Ntuli

**Affiliations:** 1 Department of Speech-Language Pathology and Audiology, Faculty of Health Sciences, Sefako Makgatho Health Sciences University, Pretoria, South Africa; 2 Department of Statistical Sciences, Faculty of Science and Technology, Sefako Makgatho Health Sciences University, Pretoria, South Africa

**Keywords:** face-to-face ABR, tele-diagnostic ABR, infant rural ABR, infant remote ABR, mobile clinic van, audiological care, tele-audiologỵ

## Abstract

**Background:**

There is a noticeable gap in access to audiology services in South Africa, and the gap is intensified in rural areas. Often, primary healthcare (PHC) facilities have an unequal ratio of audiologists to patients in need. Telehealth can expand the range of hearing healthcare services.

**Objectives:**

This study aimed to determine whether, for infants, tele-diagnostic Auditory Brainstem Response (ABR) assessment results conducted within a mobile clinic van are comparable to face-to-face diagnostic ABR results in rural Winterveldt, Pretoria North, South Africa.

**Method:**

The study utilised a quantitative, prospective cross-sectional comparative within-subject design. Each participant received both face-to-face and mobile tele-diagnostic ABR tests, which were then compared to evaluate the feasibility of mobile tele-diagnostic ABR testing. The Student’s t-test was used to determine whether there was a difference between face-to-face and tele-diagnostic tests, and Bland -Altman plots were used to assess the level of agreement between the ABR testing results.

**Results:**

There was a strong correlation (*p* < 0.001) between face-to-face and mobile tele-diagnostic ABR test results for both neurological and audiological ABR tests. The study found that there was no statistical significance between face-to-face and tele-diagnostic ABR measures; additionally, the results were within clinically acceptable and normative measures.

**Conclusion:**

Tele-diagnostic ABR offered within a mobile clinic van is feasible as it produces similar and clinically acceptable results when compared to the traditional assessment method.

**Contribution:**

This feasibility study is a positive indicator that tele-diagnostic ABR testing through a mobile clinic van may be considered to accelerate the delivery of hearing healthcare services to the infant population in rural communities.

## Introduction

Newborn hearing loss is a major health problem and the most common congenital sensory disorder (Choe et al., 2023; Guven, 2019; Sheffield, 2019) occurring in approximately 1.5 cases per 1000 live births worldwide (Choe et al., 2023). Hearing loss incidences among infants in sub-Saharan Africa may still be underrated as population-based research is insufficient (Louw et al., 2018). A study conducted by Louw et al. (2018) from two primary health facilities (PHC) located in the rural areas of Tshwane (Pretoria), South Africa resulted in an estimated hearing loss of 17.5% ranging from 3 to 97 years. And the most recent report issued by the Western Cape Government in the year 2020 stated that South Africa has an estimated 4 million deaf and hard of hearing people (Khoza-Shangase, 2022).

Telehealth services offer provision of healthcare to people in diverse contexts including those in geographically remote areas (Dimer et al., 2020; Fernandes et al., 2020). Telehealth services can also mitigate troubles associated with the lack of practitioners in far-off areas (Lima et al., 2021; Tomines, 2019). Furthermore, when faced with epidemics and pandemics such as the coronavirus 2019 (COVID-19) pandemic, containing transmission may require deviation from in-person or face-to-face clinical practice, therefore requiring the application and implementation of telehealth services (Hoi et al., 2021; Saunders & Roughley, 2021).

In audiology, the telehealth modality facilitates a variety of assessments across hearing screening, follow-up Auditory Brainstem Response (ABR) examinations, behavioural audiometry, cochlear implant programming and intervention (Hatton et al., 2019; Molini-Avejonas et al., 2015; Swanepoel & Hall, 2010). Bhamjee and colleagues (2022) found that tele-audiology in South Africa’s public healthcare system increased as a result of the COVID-19 pandemic, in that prior to the pandemic, only 7.2% reported using hearing healthcare (via tele-audiology), but nearly 19.6% used it during the COVID-19 pandemic. However, in a developing context such as South Africa, decisions regarding an on-site facilitator such as a community health worker (CHW) and the nature of training required and connectivity may have implications for policies and regulations (Khoza-Shangase, 2022).

Despite technological developments, hearing healthcare is still inaccessible to a huge population of South Africans and congenital hearing loss continues to be detected late. This largely arises from a lack of access to appropriate technology to screen or assess and diagnose infant hearing. There is a need to do further research into the feasibility of implementing tele-audiology in the South African context, hence the present study.

Technology may be used to improve strategies in the provision of healthcare particularly in the public health sector (Caetano et al., 2020; Munoz et al., 2021). Tele-diagnostic ABR offered within a mobile clinic van through the use of synchronous modalities can bridge the hearing healthcare service gap when infants are serviced remotely. This is to initiate early intervention, thereby potentially reducing the adverse effects of hearing loss.

Auditory Brainstem Response is an objective diagnostic test for assessing the auditory pathway and it is fundamental in the identification of hearing loss in infants and children who are not able to actively cooperate in providing behavioural or subjective responses for pure tone audiometry assessments (Avlonitou et al., 2011). The ABR is recorded on individuals through the use of electrodes positioned on the scalp, mastoid and to any other area inclusive of the contralateral mastoid, forehead or vertex (Plack et al., 2016). It is conducted with frequency specific stimuli and levels of its presentation are estimated according to the individual’s hearing thresholds (Harlor & Bower, 2009). The ABR is considered a good test for measuring the degree of hearing loss on individuals who cannot be tested using conventional audiometric methods such as in newborns (Plack et al., 2016). Diagnostic ABR testing is an ideal method to ensure early detection and intervention of hearing loss among infants. It is an important aspect of early detection and intervention programmes aimed at identifying and promptly treating hearing loss in infants (Ameyaw et al., 2019). The ABR is considered a good test for estimating hearing loss on individuals who cannot be tested using conventional audiometric methods (Hang et al., 2015; McCreery et al., 2015; Plack et al., 2016); and it offers results that are sufficient to permit therapeutic treatment including hearing aid fitting (Hang et al., 2015; Harlor & Bower, 2009; McCreery et al., 2015).

Access to ABR testing is a challenge in the African context as ABR equipment can be costly. This implies that ABR testing services are mostly available at tertiary hospitals. Access to these services is therefore challenging for parents living in remote and rural contexts. One of the modalities to ensure access to diagnostic ABR services is through synchronous mobile telehealth. Using mobile health clinics that are equipped with ABR facilities for testing can ensure that newborns are timeously evaluated and referred to for further management. However, there is limited evidence describing the outcomes of such a service within the South African context. Tele-diagnostic ABR services must be feasible and its results must be comparable to traditional model of ABR testing.

A few studies have been carried out to evaluate the feasibility of tele-diagnostic ABR testing. These studies revealed that data obtained through tele-diagnostic ABR, and the conventional face-to-face model of testing showed a strong correlation and the wave latencies in both methods were within the clinically acceptable range of variation (Dharmar et al., 2016; Hatton et al., 2019; Hayes, 2012; Ramkumar et al., 2013; Towers et al., 2005). However, a majority of these studies were conducted in developed contexts, thus making it difficult to replicate these findings within lower income contexts such as South Africa. Although all of the aforesaid studies indicate that tele-diagnostic ABR results were comparable to conventional face-to-face model of testing, there are evidence-based gaps regarding feasibility studies based in developing contexts and only one was carried out with a mobile clinic van. Therefore, the aim of the study was to determine if mobile tele-diagnostic ABR results in infants are comparable to face-to-face ABR results for patients accessing care at PHC clinics within the Winterveldt, Pretoria North, South Africa.

## Research methods and design

### Design and sampling

A quantitative approach was taken in this study. The study was conducted using a prospective cross-sectional comparative within-subject design. A within-subject design allowed participants to be subjected to multiple tests, and the validity of those tests did not depend entirely on randomisation. Therefore, the study aimed to evaluate all infants enrolled in the study by conducting two assessment tests on them, namely: A standard conventional diagnostic ABR test and a mobile synchronous tele-diagnostic ABR test while each subject was serving as a control for themselves, meaning that each participants’ face-to-face results were compared with their remote tele-diagnostic test results.

### Study setting

The project was conducted in three PHC clinics within the rural areas of Winterveldt, South Africa. Selection of the clinics was based on permission obtained from the managers of the various sites.

### Study population

The population was inclusive of all infants receiving care at PHC facilities in Winterveldt. This was based on caregivers who volunteered to have their infants’ auditory pathways examined through an ABR assessment.

### Sampling of participants

A total of 40 infants (80 ears) participated in the study. Participants were sampled randomly through non-probability sampling. Cappelleri and colleagues (2014) recommend a minimum of 30 subjects to evaluate the validity of a quantitative variable. The basis for participation in this study was on the subjects’ availability and willingness to do so. Infants were aged 3 days to 6 months, with or without risk factors of hearing loss. Each participant underwent a face-to-face ABR test in each year with each tracing repeated for consistency and reliability of testing. The same was followed for the synchronous ABR assessment.

### Data collection procedures

Each of the 40 infants underwent a traditional face-to-face and synchronous diagnostic ABR testing. The face-to-face ABR testing was performed in a soundproofed environment at a nearby hospital, and the tele-diagnostic ABR testing was conducted in a mobile clinic van parked just outside the PHC clinic. A CHW was used in the study to prepare patients inside the mobile clinical van. The CHW underwent orientation and training. The CHW training consisted of the following: How to give instructions to the patient; how to prepare the patient (skin preparation, electrodes placement and insert earphones); how to set up the equipment; and a mock session was conducted as a practice session with the CHW. The PATH Medical Sentiero Advanced (ABR and ASSR) system was used to assess the ABR to sound. The system was calibrated 2 months prior to the commencement of the study. Two laptops (one with the CHW inside the mobile van and one with the researcher at a nearby hospital) were connected and synchronised via the TeamViewer App. Foam tips inserted in the ear canals were used for air conduction testing. Electrodes were placed on the high forehead, pre-auricular area and the occipital nape to record brain activity in response to clicking sounds and tones that go through foam tips (earphones). The equipment only allowed the researcher to start the test post verification of electrode conductance and normal impedance levels. Table 1 shows air conduction ABR protocol utilised in this study.

**TABLE 1:** Paediatric neurological and audiological Auditory Brainstem Response norms.

Wave aspect	Wave	Mean	s.d.
Neurological ABR (80 dBnHL)	I	1.59	0.171
	V	6.253	0.321
Interpeak latency differences	I–III	2.523	0.215
	III–V	2.128	0.215
	I–V	4.653	0.287
Audiological ABR	Wave V	Mean	s.d.
	60 dBnHL	6.734	0.331
	40 dBnHL	7.426	0.358
	20 dBnHL	8.717	0.526

*Source:* Department of Speech Language Pathology and Audiology, Sefako Makgatho Health Sciences University: Electrophysiology Test Protocol, ABR, ASSR and OAE Clinical Rotation

ABR, Auditory Brainstem Response; ASSR, Auditory Steady-State Response; OAE, Otoacoustic Emission.

**FIGURE 1:**
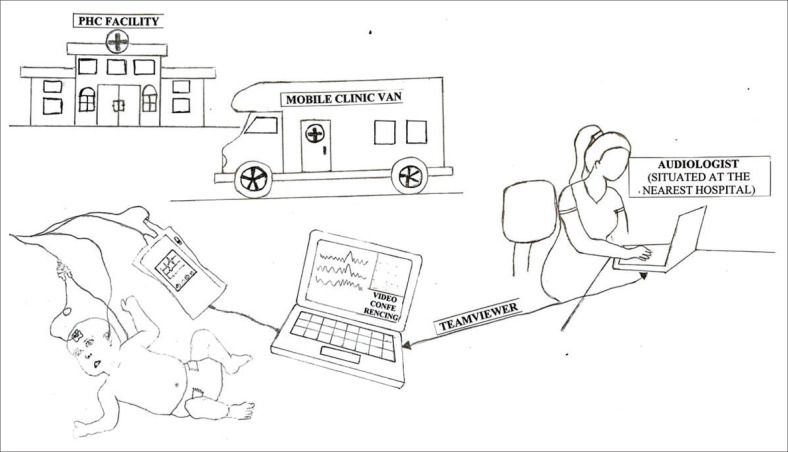
Data collection process.

A CHW was in the mobile clinic van preparing the infants for a synchronous tele-diagnostic ABR testing while the audiologist joined the session simultaneously via software Teamviewer. The audiologist at a nearest hospital connected via TeamViewer software for mirroring the test process, controlling the equipment (testing the participants) and videoconferencing. Each infant underwent both the conventional and tele-diagnostic mode of testing. The order of testing was randomised to avoid order effects. The researcher conducted testing in a counter-balanced manner in the sense that the researcher started with in-person testing for the first patient and then tele-diagnostic testing followed and the reverse occurred for the next patient. This pattern continued to ensure randomisation. Mobile tele-diagnostic ABR testing was conducted by the audiologist (researcher) and required the assistance of a CHW in the clinic van to prepare the infants for testing. Two laptops were used: The laptop in the clinic van (where there was a CHW and a caregiver) formed part of the PATH Medical Sentiero Advanced (ABR and ASSR) system and used Videoconferencing (TeamViewer installed) for the audiologist to test the infants remotely, monitor and guide the CHW. This laptop was charged with a portable power supply. The second laptop was with the audiologist in the PHC facility to test the infants as it mirrored the laptop in the mobile clinic van. Figure 1 displays an illustration of the data collection process.

**FIGURE 2:**
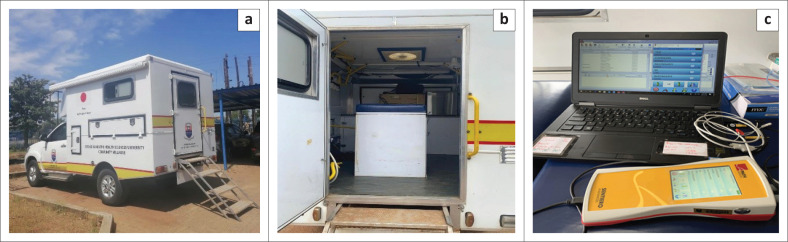
Photographs of (a) the mobile clinic van, (b) the bed in the mobile clinic van, and (c) the diagnostic Auditory Brainstem Response equipment.

The mobile clinic van contained a patient bed and a chair for testing, electricity supply and sufficient lighting, as shown in Figure 2.

### Data analysis

A descriptive statistical analysis was used to analyse and summarise results of individual or each tests (face-to-face and tele-diagnostic); this was inclusive of frequencies and percentages. Furthermore, measures of central tendency that is mean, median and mode were used to further analyse the data descriptively. Standard deviation and standard error were also applied as measures of dispersion. Correlation analysis was administered to check if there was indeed association and correlation between face-to-face and tele-diagnostic ABR results. The student’s t-test was used to assess the difference between face-to-face and tele-diagnostic tests. Bland–Altman plots were used to assess the level of agreement between face-to-face and mobile tele-diagnostic ABR testing results. The Shapiro–Wilk and the Shapiro–Francia tests were used to test the normality of the results and if they are within the normative measures. A *p*-value of 0.05 was used to test the level of significance. Analysis of variation was used to assess the level of variation between the face-to-face and tele-diagnostic ABR results. Research data were collected and recorded systematically whilst also saving in through a computing system. Participants’ data were entered onto a Microsoft Excel document, which served as a data base. Microsoft Excel was also used for cleaning and formatting data. Data in Microsoft Excel were analysed using the latest version of the SPSS software v28.0. A statistician was accessed to assist with data interpretation and verification. A statistician was used to assist the researcher with analysing the results as well as to confirm the reliability of the analysis.

### Ethical considerations

Approval was obtained from the Sefako Makgatho Health Sciences University Research Ethics Committee (SMUREC/H/348/2022: PG; NHREC no: REC 210408-003). Managers based in PHC facilities gave the researcher permission to recruit caregivers during their infants’ postnatal care visits at the clinics. Caregivers were provided with information pamphlets and an informed consent form requesting their consent for the researchers to assess their infants hearing through an ABR test using both the traditional and synchronous methods. A total of 40 caregivers consented for their infants to participate in the research project. Anonymity was maintained during data collection and analysis.

## Results

### Demographics

**FIGURE 3:**
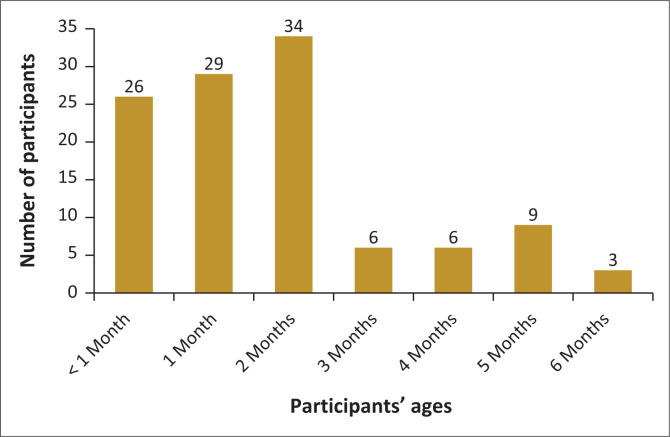
Age distribution of the infants.

A total of 40 infants (21 male and 19 female) underwent face-to-face and tele-diagnostic ABR testing, which summed to 80 ears. All participants were black African. More than two-thirds (89%) of the infants were 2 months old or less. Caregivers to the participants (80%) earned less than the minimum wage, while 20% (*n* = 8) earned above the minimum wage. The detailed distribution of the participants ages who underwent diagnostic ABR testing is displayed in Figure 3.

### Neurological Auditory Brainstem Response Assessment – Waves I, III and V (80 dBnHL)

As shown in Table 2, for Wave I, there was no statistically significant difference between the median difference of face-to-face and mobile tele-diagnostic ABR (*p* = 0.4446), and there was a strong correlation between the face-to-face and mobile tele-diagnostic ABR (*r* = 0.8450; *p* < 0.001). Similarly, Wave III showed no statistically significant difference between the median difference of the two tests (*p* = 0.0914), and the finding reported a strong correlation (*r* = 0.7441; *p* < 0.001). Again, the study result showed no statistically significant difference between the two tests for Wave V (*p* = 0.7475); however, a strong correlation was observed (*r* = 0.7356; *p* < 0.001). There was no statistically differences between the face-to-face and tele-diagnostic ABR models, Table 3 shows the interpeak wave latencies.

**TABLE 2:** A summary statistics for neurological Auditory Brainstem Response – Waves I, III and V.

Waves	Face-to-face diagnostic ABR	Mobile tele-diagnostic ABR	Mean difference
Number of ears	80	80	-
**Wave I**			
Mean	2.01	2.09	0.17
s.d.	0.78	0.82	0.49
Median	2.40	2.35	0.01
IQR	1.15	0.95	0.20
Shapiro-Francia	< 0.001	< 0.001	< 0.001
**Wave III**			
Mean	4.23	4.13	0.10
s.d.	067	0.77	0.52
Median	4.4	7.3	0.01
IQR	0.50	0.40	0.09
Shapiro-Francia	< 0.001	< 0.001	< 0.001
**Wave V**			
Mean	6.11	6.05	0.07
s.d.	0.97	1.19	0.81
Median	6.4	6.4	0.02
IQR	0.89	1.2	0.02
Shapiro-Francia	< 0.001	< 0.001	< 0.001

ABR, Auditory Brainstem Response; IQR, Interquartile range; s.d., standard deviation.

**TABLE 3:** A summary statistics for interpeak wave latencies (I-III, III-V, I-V).

Waves	Face-to-face diagnostic ABR	Mobile tele-diagnostic ABR	Mean difference
**Number of ears**	80	80	-
**Wave I and III**			
Mean	2.12	2.03	0.09
s.d.	0.50	0.53	0.38
Median	2.0	2.0	0.1
IQR	0.65	0.70	0.55
Shapiro-Francia	< 0.001	< 0.001	< 0.001
**Wave III and V**			
Mean	1.89	1.93	0.03
s.d.	0.58	0.63	0.57
Median	1.9	2.0	2.0
IQR	0.70	0.83	0.76
Shapiro-Francia	< 0.001	< 0.001	< 0.001
**Wave I and V**			
Mean	4.01	3.95	0.06
s.d.	0.75	0.88	0.65
Median	4.0	4.1	4.1
IQR	0.83	0.82	0.80
Shapiro-Francia	< 0.001	< 0.001	< 0.001

ABR, Auditory Brainstem Response; IQR, Interquartile range; s.d., standard deviation.

### Agreement between the two methods

**FIGURE 4:**
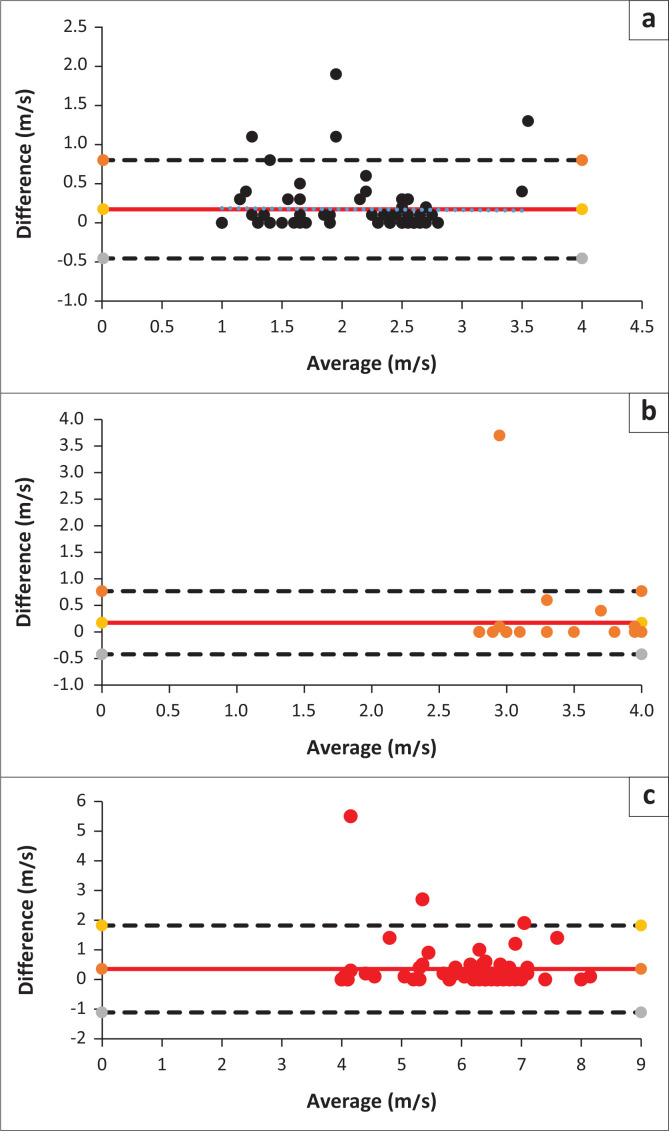
Bland-Atman plots for (a) wave I, (b) wave III and (c) wave V through face to face and mobile tele-diagnostic Auditory Brainstem Response.

Figure 4 illustrates the agreement between the two methods using the Bland–Altman technique, which shows that almost all points were within the limits of agreement for the three waves, suggesting comparability between both face-to-face and tele-diagnostic ABR measurements.

**FIGURE 5:**
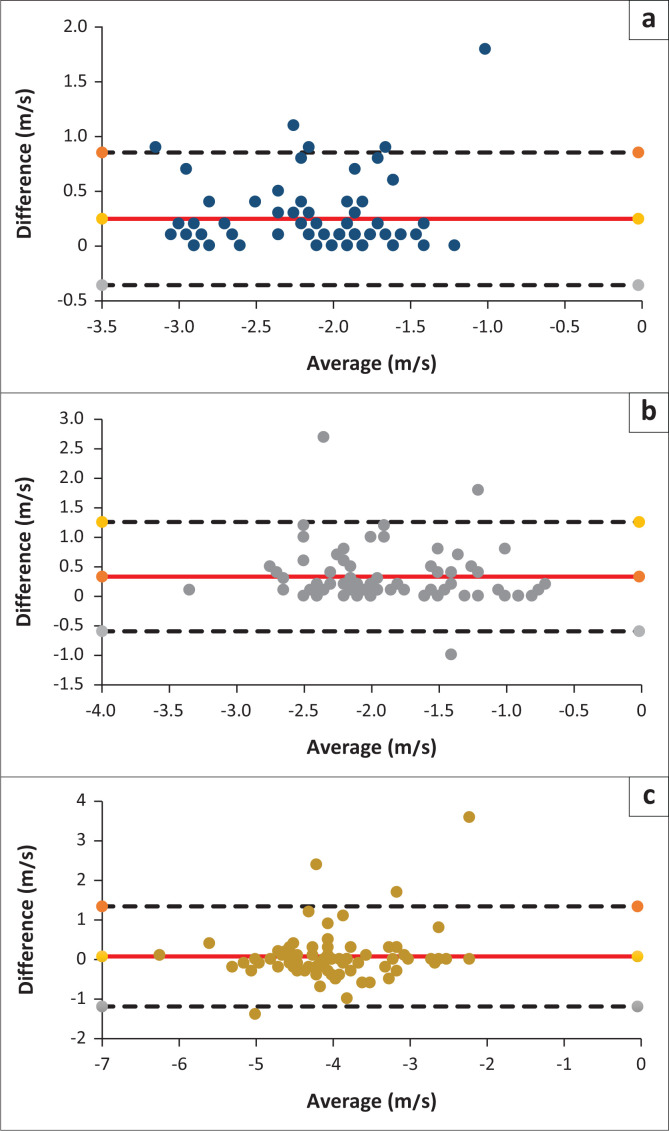
Bland-Atman plots for interpeak (a) wave I-III, (b) wave III-V, and (c) wave I-V latencies.

Figure 5 illustrates the agreement between the two methods using the Bland–Altman technique, which indicates that almost all points were within the limits of agreement for the interpeak latency differences, wave I-III, III-V, I-V, emphasising the comparability between both face-to-face and tele-diagnostic ABR measurements.

### Audiological Auditory Brainstem Response assessment

Data were analysed by using the last recognisable wave-V, meaning that the level of comparison and agreement between the last recognisable waveform (wave-V) per infant for face-to-face and tele-diagnostic ABR was measured. Wave-V was tracked to estimate the degree of hearing loss.

There was no statistically significant difference between the median difference of face-to-face and mobile tele-diagnostic ABR (8.1 vs. 8.1, *p* = 0.9125, Table 4). The study findings reported a strong correlation between the face-to-face and mobile tele-diagnostic ABR (*r* = 0.8142; *p* < 0.001).

**TABLE 4:** A summary statistics for audiological Auditory Brainstem Response – wave V.

Wave V	Face-to-face diagnostic ABR	Mobile tele-diagnostic ABR	Mean difference
Number of ears	80	80	-
Mean	7.99	7.95	0.04
s.d.	0.775	0.824	0.490
Median	8.1	8.1	0.04
IQR	0.850	0.950	0.02
Shapiro-Francia	< 0.001	< 0.001	< 0.001

ABR, Auditory Brainstem Response; IQR, Interquartile range; s.d., standard deviation.

**FIGURE 6:**
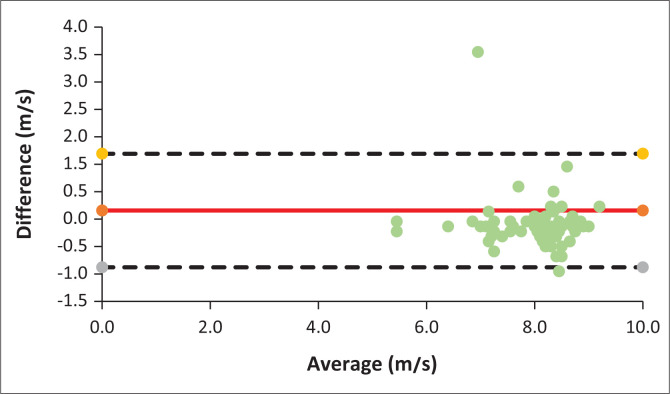
Bland–Altman Plot for Audiological Auditory Brainstem Response.

Bland–Altman’s plot illustrates that almost all points were within the limits of agreement, again suggesting no bias in the tele-measurements (Figure 6).

There was agreement between the two assessment methods. Results indicate that there was a strong correlation (*p* < 0.001) between audiological face-to-face and mobile tele-diagnostic ABR assessment results.

## Discussion

The study compared face-to-face and mobile tele-diagnostic ABR test results in infants and found them comparable. This feasibility was demonstrated through the assistance of a CHW, similar to the study conducted by Ramkumar and colleagues (2013) where a village healthcare worker was in a mobile clinic van to prepare and monitor the infant for real-time tele-diagnostic ABR testing. This study’s findings indicated no statistically significant difference between face-to-face and tele-diagnostic ABR testing, and the results were within clinically acceptable and normative measures.

The findings of the study are like that of published studies. Ramkumar and colleagues (2013) showed that the latency results for synchronous tele-diagnostic ABR tests were similar to conventional face-to-face diagnostic testing, with a strong correlation between both models of hearing healthcare services. Towers and colleagues (2005) also revealed that there was no statistically significant difference between the two methods of testing and the wave latencies in both procedures were almost similar. Dharmar and colleagues (2016) reported that diagnostic ABR testing was performed successfully through real-time synchronous measures, with the assistance of a trained facilitator. Hatton and colleagues (2019) and Hayes (2012) also reported the same regarding the success of real-time testing methods.

The findings of this study suggest that diagnostic ABR testing can be conducted from a mobile clinic van using a CHW to set up the patient. This has benefits for early detection and intervention by increasing service delivery to patients in rural and remote areas. Considering the strain on financial, human and technical resources faced by the South African healthcare sector, telehealth services can expedite taking specialist services to communities with limited access. This promotes the implementation of Early Hearing Detection and Intervention (EHDI), which is well documented across the literature (Graydon et al., 2019; Kanji & Khoza-Shangase, 2021; Mostafa et al., 2022; Yoshinaga-Itano, 2004; Yoshinaga-Itano et al., 2017). In addition, hearing loss (sensory deficit) is reportedly a remarkable epidemiologic burden (Nocini et al., 2023); thus, early detection will increase early intervention, reducing its contribution to the global burden of diseases.

Auditory Brainstem Response testing is an objective test that can be used to obtain reliable audiological information on infants (Khaimook et al., 2019; Mattiazzi et al., 2023). As a result of the skill set required to conduct an ABR assessment and the cost of equipment, ABR services are not readily available. These challenges hamper the progress of early detection and timeous intervention of hearing loss (Bezuidenhout et al., 2018; Gina et al., 2021). The use of telehealth-based diagnostic services can mitigate these challenges. Furthermore, the use of synchronous telehealth services can widen access to hearing healthcare services. Telehealth services can potentially bring developing countries closer to the goal of the Universal Newborn Hearing Screening (UNHS) programme, through bringing objective hearing assessments closer to the patient. This type of service delivery promotes the model of decentralised care: a model of much-needed contexts where access to healthcare services is limited (Komalasari, 2023; Ncube et al., 2023).

There were many benefits in offering the service from a mobile clinic van. This set-up meant that the service could be moved around according to need and challenges (impact of noise). In a country where space and fixed infrastructure is limited, the use of mobile clinic vans can be practical, efficient, and cost effective (Ashwood et al., 2017; Schnippel et al., 2015). Remote healthcare has multiple benefits for ‘at home’ (direct-to-patient) services and a capacity to save costs and improved access to audiological services (Ashwood et al., 2017; Aung et al., 2015). The mobile clinic van was comfortable and well-resourced with a sufficient electricity supply. With regard to the challenges related to environmental noise – a recommendation would be to consider sound treating mobile clinic vans, as they are more conducive to audiological testing.

The study confirmed that tele-diagnostic testing can be successfully conducted by using a well-trained CHW to prepare and set up the patient. Studies indicate that CHWs are essential in promoting health and healthcare services (Murphy et al., 2021; Smithwick et al., 2023). Community health workers also serve as important links between communities, facilities and services (Kok et al., 2017; Murphy et al., 2021; Smithwick et al., 2023).

The study’s findings revealed that offering tele-diagnostic ABR services from a mobile clinic van for infant hearing healthcare is feasible. Technological advancements can have a positive impact in the healthcare systems (Bhavnani et al., 2016; Vishwakarma et al., 2023). They enable practitioners to provide services to underserved populations, improving access to auditory care services for infants.

### Strengths and limitations

The strength of the study is that it was conducted in a mobile clinic van, set-up just outside a PHC clinic and positioned in an area accessible to patients seeking postnatal care services. This improved access to services for patients living in rural and remote areas. This also implies that EHDI programmes can be enhanced by using synchronous telehealth-based models to bring services closer to communities, thereby mitigating the global burden of hearing loss. Furthermore, the mobile nature of the clinic van provided flexibility as it could be parked or positioned in more convenient spaces and quieter environments.

The small sample size limits the extent to which the results are generalisable to broader populations. Minor difficulties relating to internet connectivity were experienced during synchronous tele-diagnostic ABR testing (data collection period). This occasionally caused poor video and/or visual images quality when observing the infant while testing and poor audio quality during feedback sessions with the caregiver to the infant.

In this study, an attempt was made to minimise examiner-related variables by training the CHW prior to the pilot study and fully involving them during pilot study; however, it is almost or practically impossible to completely eliminate the human factor. It goes without saying that the practice of remote hearing healthcare is not immune to such factors. To give an example, placement of electrodes and insert earphones on infants was conducted by a CHW; therefore, there may have been some variability in participants’ results. In this study, remote ABR was conducted synchronously through the assistance of the CHW. The study found that the CHW holds important value for community-based healthcare programmes and can bridge the gap between the patient and their access to specialist services. This has future implications for improved health outcomes in terms of patient support, education and orientation to healthcare technology and resources.

Synchronous tele-diagnostic ABR services provided through the assistance of a CHW offer opportunities to deliver hearing health services to patients residing in remote areas. Asynchronous ABR can be utilised considering that the model obtains the same quality as in-person visits as concluded in this study.

### Recommendations

Larger samples are required for future studies. A preliminary survey of audiologists’ views, opinions and experiences concerning tele-diagnostic ABR testing offered within a mobile clinic van should be conducted. It also requires stakeholder’s awareness to bridge the gap between knowledge and action. Lastly, a comprehensive cost-benefit analysis of tele-diagnostic ABR offered within a mobile clinic van in the healthcare sector would be of benefit.

## Conclusion

The results of the study indicate that tele-diagnostic ABR offered within a mobile clinic van is feasible as it produces similar and clinically acceptable results when compared to the conventional method of testing. Telehealth-based service delivery has some challenges, such as the need for a strong and reliable internet connectivity signal when conducting tests. This study was conducted in a mobile clinic van, set up next to the clinic and positioned at a site accessible to caregivers and infants attending postnatal care in the facilities. This is associated with ‘at home’ (direct-to-patient) telehealth, its ability to reduce costs, as well as eliminating the need for many socioeconomically disadvantaged caregivers to travel long distances to hospitals. This article contributes to the field of tele-audiology by providing ideas that may impact future hearing health services, including the potential of asynchronous testing through assistance by CHW or an on-site facilitator.
